# Case of Traumatic Tetanus Cured by the Inhalation of Chloroform

**Published:** 1853-07

**Authors:** Th. V. Dusch

**Affiliations:** Mannheim


*Case of Traumatic Tetanus cured by the inhalation of Chloroform.* By Dr. Th. V. Dusch, of Mannheim.—A patient, aged 26, ran a nail into the great toe of the left foot, on April 17th. On the 30th, he experienced difficulty in opening the mouth. On May 2d, the masseter and other masticatory muscles were contracted, and the mouth was closed. On the 5th, the symptoms were more severe; and on the 6th, there were tetanic spasms, returning every five minutes, combined with opisthotones. Morphine, bleeding, tobacco enemata, cupping, etc., were tried without avail. Pulse 112; skin warm. Chloroform inhalations were then directed. The tetanic symptoms disappeared during the narcotism, and the patient obtained some rest. Upon the return of the spasms, the chloroform inhalations were repeated, during which the pulse sank from 110 to 60. The author now endeavored to keep the patient in a state of constant excitement, and administered, between this date and 20th of May, 64½ ounces of chloroform.

21st.—The inhalations were discontinued; cold was applied to the shaven head, and a quarter of a grain of acetate of morphia was given every two hours. On the 4th of June the patient was well.

Dr. Bargigley of Lesbos, relates the case of a countryman, in whom tetanic symptoms came on after the ex-articulation of the third finger. The patient was cured by the same treatment.

A similar case is related in Langenbeck's Clinic.

The death of the patient in tetanus is caused by the cramps extending to the respiratory muscles and to the heart. It rarely occurs from loss of power in the brain or spinal cord, or from exhaustion. The course is usually acute, the tetanic symptoms ensuing in the following order:—trismus, cramp in the muscles of back and neck, the extremities often remaining free. Most patients die before the twelfth day. The prognosis is the more favorable the longer the disease lasts. The advantage of narcotising remedies consists in this, that the tetanic cramps become suppressed or rendered milder, and thus the respiratory act and the contractions of the heart proceed unimpaired. Chloroform, from being taken into the blood direct from the lungs, is preferable to all other narcotising remedies, which must be absorbed from the stomach. The effects of the chloroform inhalations must be continuous, the patient remaining days, and even weeks, more or less, under its influence, until a point of saturation is attained, characterized by the rapid production of the extreme narcotising effects, the patient during the intervals being in a state of obstinate excitement; head hot, face red, eyes glistening, frequent epistaxis, etc. Atmospheric air was mixed with the chloroform in Dr. Dusch's case.—*Med. Times and Gaz.* from *H. und Pfs. Ztschr., N. F.* III. 1852.

